# Traditional Chinese medicine reimbursement reform in China: implementation progress, policy constraints, and optimization strategies

**DOI:** 10.3389/fpubh.2026.1923879

**Published:** 2026-09-03

**Authors:** Fazheng Zhao, Yichi Chen, Xin Tong

**Affiliations:** 1 Zhejiang Cultural Research Institute of Chinese Medicine, Zhejiang Chinese Medical University, Hangzhou, China; 2 College of Humanities and Management, Zhejiang Chinese Medical University, Hangzhou, China

**Keywords:** DRG payment, health insurance payment reform, reimbursement policy, traditional Chinese medicine, value-based payment

## Abstract

**Introduction:**

As the inheritance, innovation, and development of TCM advance, medical insurance reimbursement policies are intended to improve access, guide service structure, and support high-quality development. This study examines the implementation progress, policy constraints, and optimization paths of TCM reimbursement reform in China.

**Methods:**

The study analyzes publicly available national and regional policy documents, together with secondary literature, to synthesize the policy design, implementation arrangements, and evidence boundaries of TCM reimbursement reform.

**Results:**

The policy corpus suggests that national and local governments have progressively expanded insurance support for eligible TCM institutions, techniques, and decocted herbal medicines, creating institutional conditions for broader reimbursement arrangements. At the implementation level, some regions have explored TCM outpatient syndrome-differentiation fees, DRG-based incentives, and outpatient global-budget management. However, current payment rules still do not fully align with TCM diagnostic and treatment characteristics. Key issues include incomplete recognition of syndrome differentiation and treatment value under DRG payment, low TCM service prices, insufficient reimbursement levels, static control of TCM decoction pieces costs, tension between zero-markup policies and payment reform, and immature quality-evaluation mechanisms under centralized procurement.

**Conclusion:**

Based on these findings, the study proposes refining TCM-specific payment and pricing mechanisms, adjusting disease grouping and payment rules, dynamically revising cost-control standards, coordinating markup reform with compensation policies, and developing quality grading and “higher quality, higher price” mechanisms for herbal materials. Because the evidence is primarily policy-level, the study identifies policy instruments and implementation pathways rather than causal effects on service utilization, patient access, fund expenditure, or clinical outcomes.

## 1 Introduction

With the public’s growing health awareness and increasing recognition of traditional medicine, TCM-with its unique theoretical framework, diverse therapeutic methods, and deep cultural heritage-is playing an increasingly prominent role in disease prevention, treatment, rehabilitation, and health management. TCM is not only an integral part of China’s healthcare system but also a vital resource for advancing the “Healthy China” initiative (1, 2). As a key institutional tool linking the supply of medical services, patients’ ability to pay, and the operational efficiency of health insurance funds, health insurance reimbursement policies are intended to shape the conditions for developing TCM services. From the perspective of national policy implementation, medical insurance support for the inheritance, innovation, and development of TCM has become a key direction for both national and local healthcare reform (3, 4). Through measures such as expanding the scope of medical insurance coverage, improving service pricing management, exploring diverse payment methods, and strengthening fund performance management, the medical insurance system is expected to create conditions under which policy support may be translated into more favorable arrangements for TCM services, although the effects require empirical verification. However, TCM diagnosis and treatment are characterized by syndrome differentiation and treatment, individualized therapy, holistic regulation, and long-term intervention. Existing medical insurance reimbursement rules are still largely based on Western medical diagnostic models and modern medical coding systems, making it difficult to fully recognize and reasonably compensate for the value of TCM services. Therefore, building on an assessment of policy implementation progress and evidence boundaries, further analyzing the practical challenges in the current reform process, and proposing targeted optimization strategies are urgent tasks that must be addressed at present.

## 2 Methods

This study adopted a qualitative policy document analysis design. The objective was not to estimate the causal effect of a single payment policy, but to synthesize the institutional logic, implementation progress, and policy constraints of TCM reimbursement reform in China. The analysis combined national policy documents, provincial and local implementation materials, and peer-reviewed literature on health insurance payment reform, DRG payment, centralized procurement, and TCM service delivery. No internal health insurance settlement data, provider-level operational data, or patient-level outcome data were available to this study; therefore, claims about implementation progress are based on documented policy instruments and implementation arrangements rather than measured causal effects.

### 2.1 Policy document sources and search strategy

Policy materials were identified from publicly available official sources, including the National Healthcare Security Administration, the National Health Commission, the National Administration of Traditional Chinese Medicine, provincial healthcare security administrations, and local government or health insurance authority portals. Searches focused on policy documents issued from 2016 onward, when the legal and institutional framework for TCM inheritance and innovation became more closely linked with health insurance payment reform. Search terms included Traditional Chinese medicine, medical insurance, reimbursement, payment method reform, DRG, DIP, outpatient global budget, TCM service price, Chinese herbal medicine, decoction pieces, centralized procurement, and quality evaluation.

The policy search and source verification were conducted between August 4 and August 6, 2026, and the search was last updated on August 6, 2026. The final referenced corpus comprised 23 sources: 13 peer-reviewed secondary studies on TCM service delivery, health insurance payment, zero-markup reform, centralized procurement, and herbal medicine quality governance; and 10 publicly available official policy or government information sources used as the core policy corpus for national and regional analysis (5–14). These official sources covered national guidance and selected provincial or municipal implementation materials from Zhejiang, Shanghai, Guangdong/Jiangmen, and Jiangsu/Nanjing.

### 2.2 Policy selection criteria

Documents were included if they met all of the following criteria: (1) they were formal laws, regulations, policy opinions, implementation plans, notices, catalogues, or technical guidance issued by national ministries, provincial governments, or health insurance authorities; (2) they were directly related to TCM reimbursement, health insurance payment reform, DRG or DIP payment, pricing of TCM medical services, reimbursement of Chinese herbal medicines or decoction pieces, centralized procurement, or quality governance; (3) they contained substantive information on policy objectives, payment rules, coverage arrangements, price compensation, cost-control mechanisms, provider incentives, or implementation pathways; and (4) they were publicly accessible through official channels or cited academic sources.

Documents were excluded if they were general news reports, media summaries without explicit policy instruments, opinion articles, conference notices, duplicate releases of the same policy text, or materials concerned only with general cultural promotion of TCM without a direct link to health insurance payment, reimbursement, pricing, procurement, or service coverage. National-level policies were used to identify the overall reform direction, while provincial and local documents, with Zhejiang as an illustrative focal case, were used to examine implementation mechanisms and regional policy constraints.

For the regional comparison, Zhejiang was treated as a focal case because the province has issued explicit TCM-related outpatient budget management and DRG incentive arrangements. Shanghai, Guangdong, and Jiangsu were selected as comparator regions because their official documents provide clear examples of value-oriented pricing, disease-based payment, DRG/DIP adaptation, and quality governance for TCM. The comparison therefore represents purposive policy sampling rather than exhaustive provincial coverage, and it is designed to compare policy instruments rather than to estimate cross-regional causal effects.

### 2.3 Analytical framework

The selected materials were analyzed through a structured framework covering five dimensions: payment rules and grouping logic, reimbursement coverage and benefit design, price compensation for TCM services, cost-control and provider incentive mechanisms, and quality governance for Chinese herbal medicines and related procurement. These dimensions were used to organize the subsequent discussion of implementation progress, policy constraints, and optimization strategies. Because the study is based primarily on policy texts and secondary literature, the findings should be interpreted as a policy analysis rather than a quantitative impact evaluation.

For each included official source, the extracted fields included issuing authority, jurisdiction, year of issue, policy level, payment or pricing instrument, TCM-specific coverage or management rules, provider incentive or cost-control mechanism, disclosed quantitative, operational, or outcome indicators, and the boundary of the available evidence. The analysis proceeded in four steps: first, national policy direction and regional implementation materials were screened against the inclusion and exclusion criteria; second, eligible policy instruments were coded into the five analytical dimensions; third, regional differences were summarized in a comparison matrix; and fourth, numerical indicators were reported only when they were explicitly disclosed in official or public sources, with unavailable outcome indicators identified rather than imputed.

## 3 Implementation progress of health insurance reimbursement policies for traditional Chinese medicine

In recent years, the central government has successively issued policy documents such as *the Law of the People’s Republic of China on Traditional Chinese Medicine*, *the Opinions of the Central Committee of the Communist Party of China and the State Council on Promoting the Inheritance and Innovative Development of Traditional Chinese Medicine*, *the Several Policy Measures on Accelerating the Distinctive Development of Traditional Chinese Medicine*, and *the 14th Five-Year Plan for the Development of Traditional Chinese Medicine*, providing an institutional foundation for reforms in medical insurance reimbursement for traditional Chinese medicine. In 2021, the National Healthcare Security Administration and the State Administration of Traditional Chinese Medicine issued the “Guiding Opinions on Medical Insurance Support for the Inheritance and Innovative Development of Traditional Chinese Medicine,” which explicitly stipulated that eligible TCM service providers be included in the medical insurance designated provider management system, that price management for TCM services be improved, and that eligible TCM decoction pieces, proprietary Chinese medicines, TCM preparations produced by medical institutions, and appropriate TCM techniques be included in the scope of medical insurance coverage in accordance with regulations.

In terms of implementation progress, first, the policy support system for TCM under medical insurance has been gradually refined in formal policy design. The national “14th Five-Year Plan for the Development of Traditional Chinese Medicine” explicitly calls for improving TCM pricing and medical insurance policies, establishing a health technology assessment system for TCM medical services that aligns with the characteristics of TCM, and optimizing pricing policies for TCM medical services. These national-level arrangements provide the institutional basis for local governments to translate general policy guidance into more specific payment, pricing, and provider-management rules, although their effects on providers and patients still require empirical verification.

Second, the documented scope of coverage for TCM services has been broadened in policy design. At the national level, there is an emphasis on supporting the inheritance and innovative development of TCM through medical insurance. Eligible TCM medical services and herbal medicines are being incorporated into the basic medical insurance coverage in accordance with established procedures, and efforts are underway to explore medical insurance payment methods tailored to the characteristics of TCM. This specifically encompasses aspects such as price management for TCM services, multi-stakeholder coordination of TCM medical insurance policies, reform of medical insurance payment methods, and management of the medical insurance reimbursement directory. These arrangements are intended to provide financial support for TCM services and to create conditions for potential improvements in service quality and efficiency, which still require verification through outcome data. For example, including fees for TCM outpatient syndrome differentiation and treatment within the scope of health insurance coverage is intended to lower financial barriers to TCM outpatient services. However, its effects on service utilization, patients’ willingness to seek TCM care, patient financial burden, and provider behavior require verification using claims data, provider-level records, or patient-level data. The current evidence therefore supports claims about coverage expansion and policy design rather than measured changes in access, affordability, or utilization.

At the regional level, Zhejiang provides a particularly relevant focal case. Its policy on improving TCM medical insurance payment specified an outpatient global-budget arrangement for TCM decoction-piece services under which surpluses could be retained and overspending shared; it also included TCM outpatient syndrome-differentiation and treatment fees in fund coverage, supported contracted “Internet+” TCM services for designated institutions, and established inpatient DRG incentives linked to TCM treatment. More recent Zhejiang DRG management procedures further incorporated TCM subgrouping and quality-linked indicators, including the average cost of inpatient decoction pieces. Together, these measures indicate a policy-design shift from general reimbursement inclusion toward explicit budget, pricing, and DRG incentive design (6,7).

To move beyond a single-case description while avoiding unsupported claims about effects, Table 1 compares Zhejiang with selected national and regional policy examples. The comparison treats official policy instruments and explicitly stated implementation arrangements as observable policy indicators, rather than as causal outcome estimates.

**Table 1:** Regional policy evidence on TCM reimbursement reform in selected Chinese jurisdictions.

Jurisdiction/source	Payment or pricing instrument	TCM-specific policy indicator/evidence	Analytical implication
National baseline NHSA and NATCM guidance, 2021 (5)	Global budget adjustment; DRG/DIP adaptation; disease-based, bed-day, and capitation payment options.	Eligible TCM institutions, services, preparations, decoction pieces, and appropriate techniques may be incorporated into designated provider management and insurance coverage.	Creates the policy mandate for local adaptation rather than a single national payment formula; this is policy-level evidence rather than an outcome estimate.
Zhejiang TCM payment policy, 2022; DRG procedure, 2025 (6, 7)	Outpatient global budget; surplus-retention and overspending-sharing rules; inpatient DRG incentives; TCM subgrouping.	Links TCM outpatient syndrome-differentiation fees, decoction-piece cost control, designated online TCM services, and inpatient DRG incentives to fund management.	Documents a policy-design case for translating TCM value into provider-level budget and DRG incentive design; provider- or patient-level effects require claims or institutional data.
Shanghai Municipal measures, 2021 (8)	Dynamic TCM service price adjustment; disease-based payment; DRG/DIP inclusion; quality grading and traceability.	Emphasizes TCM advantageous diseases, value-oriented pricing, quality management for decoction pieces, and payment arrangements linked to clinical-value considerations.	Combines price compensation, payment reform, and herbal medicine quality governance; available evidence mainly documents formal instruments.
Guangdong Payment reform guidance, 2021 (9)	Outpatient disease-based payment; inpatient disease-score payment; TCM day-ward payment; family-doctor TCM packages.	Selects TCM advantageous diseases and explores disease-specific payment and capitation-linked primary-care TCM service packages.	Highlights disease-specific and primary-care-oriented experimentation; patient utilization and expenditure effects are not estimated here.
Jiangsu Implementation opinion, 2022 (10)	DRG disease grouping; DIP point adjustment; single-disease payment; same-disease, same-effect, same-price principle.	Allows DRG regions to explore TCM-specific groups and value-oriented or effectiveness-informed payment, while DIP regions may increase points for TCM advantageous diseases.	Documents adaptation across DRG, DIP, and single-disease payment settings; causal effects on fund expenditure or care quality remain outside this study.

This table summarizes policy-level evidence from official documents. It identifies reimbursement and payment mechanisms and clarifies the boundaries of the available evidence; it does not estimate causal effects on provider behavior, patient access, fund expenditure, or clinical outcomes. Source: compiled by authors based on official policy documents from official government websites.

The regional comparison indicates that TCM reimbursement reform in China is not proceeding through one uniform model. Zhejiang emphasizes fund-budget incentives and DRG adaptation, Shanghai combines pricing and quality governance, Guangdong relies more on disease-specific and primary-care payment experiments, and Jiangsu adapts TCM incentives across DRG, DIP, and single-disease payment settings. This variation strengthens the policy relevance of the analysis while also showing why TCM reimbursement reform requires region-specific implementation rules.

To address the limited availability of quantitative evidence in publicly accessible policy documents, Table 2 reports numerical, operational, and selected municipal outcome indicators verified from official or public sources. Unavailable comparable statistics are identified directly rather than imputed.

**Table 2:** Publicly available quantitative, operational, and selected outcome indicators for selected TCM reimbursement reforms.

Jurisdiction/source	Indicator type	Publicly disclosed value
National baseline NHSA and NATCM guidance, 2021 (5)	National TCM payment guidance	No unified national coefficient, disease count, reimbursement ratio, payment standard, or outcome indicator is disclosed.
Zhejiang TCM payment policy, 2022 (6)	Outpatient budget and DRG incentive parameter	Budget calculation uses 2021 utilization/cost data; TCM DRG incentive coefficient ceiling is no more than 15%; local surplus/overspending ratios are not standardized.
Shanghai Municipal measures, 2021 (8)	Municipal reform package	The policy contains 19 measures; disease counts, payment ranges, reimbursement ratios, and outcome indicators are not disclosed.
Guangdong / Jiangmen 2021–2024 (9, 11, 12)	Provincial reform package and municipal DIP catalogue	Provincial summary describes seven TCM payment-reform areas; Jiangmen 2024 catalogue lists 80 TCM advantageous diseases and 2 TCM day-ward diseases.
Jiangsu / Nanjing 2022–2024 (10, 13, 14)	Municipal DRG grouping and selected outcome indicators	Nanjing reported that TCM DRG groups increased from 79 to 85 in 2024. Official citywide DRG reporting also showed 8.8% lower inpatient expenditure, RMB 486.11 lower average patient burden per discharge, and a 119.90% settlement rate with RMB 12.424 million surplus for TCM DRG groups.

This table reports publicly available policy parameters, operational indicators, and selected municipal outcome indicators only. It does not impute missing values and does not treat policy counts, coefficients, local disease-group numbers, or single-city results as evidence of causal effects on provider behavior, patient access, expenditure, or clinical outcomes. These publicly available indicators supplement the policy evidence but do not substitute for causal evaluation. Source: compiled by authors based on official policy documents from official government websites.

Because comparable outcome indicators were not publicly available across jurisdictions, the reported Nanjing data are used only as descriptive public evidence and should not be interpreted as causal evidence of TCM reimbursement reform.

Overall, Tables 1, 2 suggest policy-level implementation progress and limited local outcome signals rather than fully demonstrated comparable outcome effects: policy support appears more systematic in available documents, coverage rules have been broadened on paper, and local policy experimentation has become more visible. Public sources disclose selected payment parameters, local implementation indicators, and Nanjing municipal outcome indicators, such as Zhejiang’s TCM DRG incentive coefficient ceiling, Nanjing’s expanded TCM DRG grouping, reported reductions in citywide inpatient expenditure and patient burden under DRG operation, and reported settlement surplus of Nanjing TCM DRG groups. Nevertheless, these indicators remain uneven and locally specific. Therefore, the discussion is about to shift from policy progress to the constraints that may limit the translation of TCM-specific payment rules into measurable clinical and economic value.

## 4 Current status and issues in the reform of the traditional Chinese medicine health insurance system

Against the backdrop of the in-depth implementation of the “Healthy China” strategy, TCM faces tremendous development opportunities, while reforms to its health insurance payment methods are steadily advancing. Nevertheless, as social needs evolve, certain constraints have emerged in these reforms. The constraints identified below explain why policy inclusion has not automatically translated into fully realized clinical or economic value. They operate across payment rules, service pricing, prescription cost control, compensation for decoction pieces, and quality governance under centralized procurement. Since these domains are interconnected, reforms in one area may affect provider incentives, fund management, patient affordability, and the sustainability of TCM institutions.

### 4.1 DRG payment rules are not aligned with the characteristics of traditional Chinese medicine diagnosis and treatment

The current DRG payment system is not fully aligned with the TCM diagnostic and treatment model, which may pose operational challenges for TCM medical institutions (15, 16). Specifically, the payment system may not adequately capture the potential value of TCM treatment. The DRG payment system primarily classifies and reimburses based on modern medical treatment methods, and may overlook the distinctive features of TCM in syndrome differentiation and individualized treatment. TCM treatment methods are not fully reflected in the DRG grouping of inpatients, which may leave the potential therapeutic value of TCM for these patients insufficiently reflected in medical insurance payments. Furthermore, there may be a mismatch between medical insurance payments and treatment needs; outcome effects still require empirical verification. Although TCM treatments are regarded as suitable for some diseases, available policy-level evidence does not verify comparative effectiveness; because the DRG payment system does not account for the characteristics of TCM treatment, medical insurance payments may not yet provide a transparent basis for recognizing the potential health and economic value of TCM treatment.

In practice, the therapeutic value of TCM is often not fully reflected in DRG groupings and payment standards. Although TCM is regarded as having potential advantages for certain conditions, the treatment process, technical value, and potential long-term health benefits are not adequately reflected in medical insurance payments. For patients requiring rehabilitation, those with chronic diseases, and the older population, TCM treatments may be suitable; however, under per-bed-day payment or DRG management systems, insufficient incentives and compensation can dampen healthcare institutions’ enthusiasm for providing TCM-specific services. Furthermore, the insufficient weighting of factors such as secondary surgeries and combined treatments within the DRG system may also subject healthcare institutions to significant cost pressures when admitting and providing comprehensive treatment for complex cases.

### 4.2 Prices for traditional Chinese medicine services are relatively low, and health insurance reimbursement is insufficient

TCM is considered to have potential therapeutic advantages in disease prevention, treatment, and rehabilitation. However, the current share of TCM medical service costs in overall healthcare expenditures remains relatively low, reflecting that health insurance reimbursement policies still provide insufficient support for TCM services.

First, the low pricing of TCM service items affects the level of health insurance reimbursement. Taking services such as fracture reduction, acupuncture, tuina massage, and syndrome differentiation and treatment as examples, the technical expertise, consultation time, and potential therapeutic value involved are often not adequately reflected by the existing pricing mechanisms. This phenomenon highlights a possible disconnect between the pricing of TCM medical services and their clinical value, which may not fully reflect that value and may consequently limit the scope and proportion of these services covered by health insurance. Since medical insurance reimbursements are based on current pricing, TCM medical institutions are unable to secure revenue aligned with the technical and potential therapeutic value of these services, leaving them under long-term financial pressure.

Second, the structure of medical insurance reimbursement may encourage the “Westernization” of TCM medical services. Faced with the low revenue generated by TCM services, some TCM medical institutions have shifted toward offering higher-priced Western medical diagnostic and treatment services to maintain operations. This trend toward “Westernization” may further weaken the distinctive characteristics of TCM (17). High-cost Western medical treatment items may become more prominent in TCM hospitals, the current medical insurance reimbursement system may not fully account for differences in TCM’s treatment methods and potential therapeutic efficacy, which may reinforce TCM medical institutions’ reliance on Western medical services and limit the conditions for realizing the distinctive value of TCM therapies.

Third, the coverage of TCM services in the medical insurance directory is insufficient, and the pricing mechanism lags behind actual demand, constraining the provision of services. Currently, the pricing of many TCM medical services remains low, and the majority of TCM services are not covered by medical insurance. At the same time, the existing pricing mechanism has not been adjusted in a timely manner, making it difficult to fully reflect the technical complexity, potential therapeutic value, and actual costs of TCM treatments. TCM medical institutions face the problem of insufficient compensation under the medical insurance payment system, making it difficult to create conditions for sustainable operation by relying solely on TCM services. As a result, many TCM hospitals are forced to rely on Western medical treatment services to maintain basic operations, while the role of TCM treatments may be reduced in some settings.

### 4.3 There is a lack of dynamic adjustments to the cost control standards for traditional Chinese medicine decoction pieces

Under the current health insurance payment system, which is primarily based on a fee-for-service model, the static control standards for the average cost of TCM prescriptions have become a prominent issue that constrains clinical TCM diagnosis and treatment and may limit the intended effects of reforms. Some regions have long continued to use TCM prescription cost control standards established in the past, failing to make dynamic adjustments in response to changes in herbal medicine prices, labor costs, processing costs, and the disease spectrum. As a result, health insurance payments are based on these static standards, which are significantly out of line with actual costs, leading to a situation where the actual costs of essential services provided by TCM medical institutions cannot be covered.

Such strict cost controls impose significant restrictions on TCM medical institutions-such as TCM clinics-when prescribing classical formulas during specific diagnostic and treatment processes, thereby potentially limiting individualized treatment and weakening the intended policy objective of health insurance payments, which is to encourage appropriate use of TCM. Furthermore, rigid reimbursement standards restrict the clinical principle of TCM diagnosis and treatment based on syndrome differentiation, which runs counter to the value orientation of medical insurance payment reform. The uniform per-prescription cost cap fails to fully account for disease complexity, disease progression stages, and individual differences. When treating chronic and complex conditions with TCM, the cost of herbal ingredients in high-quality prescriptions that adhere to the principles of syndrome differentiation and treatment often exceeds the price cap. This results in unreasonable interference with TCM clinical practices, distorts the natural course of TCM treatment to some extent, and may affect therapeutic appropriateness.

The current reform of medical insurance payment methods aims to establish a mechanism that better reflects the value of technical services while creating conditions for cost-effectiveness and efficiency, both of which require empirical validation. However, the crude management approach of setting absolute monetary caps on TCM decoction pieces fails to reflect the principle of “higher quality, higher price” and does not by itself verify that medical institutions can reduce patients’ overall medical expenses through improved TCM-related outcomes. This runs counter to the “value-based healthcare” orientation of medical insurance payment reform.

### 4.4 The markup policy for traditional Chinese medicine decoction pieces conflicts with the direction of medical insurance payment reforms

Against the backdrop of deepening reforms in medical insurance payment methods, the markup policy for TCM decoction pieces-as a traditional compensation mechanism-appears to have developed tensions between its operational logic and the reform objectives, becoming a challenge that requires further assessment in the reform process. On the one hand, the markup policy tends to create incentives for price increases among medical institutions and suppliers; on the other hand, simply eliminating the markup could subject TCM medical institutions-which have long relied on revenue from decoction pieces-to significant operational pressure. Following the implementation of the zero-markup policy, available medical insurance settlement results suggest that the “net profit” originally derived from markups may not be converted into retained medical insurance surpluses as expected and may be offset by rising drug prices. The “net income” that TCM hospitals previously obtained through markups may therefore not be retained as expected, adding to operational pressure.

Specifically, under the TCM decoction pieces markup policy, both hospitals and suppliers tended to raise prices to secure higher markup revenue and corporate profits. At the same time, the medical insurance settlement cycle is relatively long-typically not completed until the second half of the following year-causing a disconnect between the medical insurance surplus incentive policy and the timing of monthly bonus distributions at hospitals, which may weaken the intended incentive function of the mechanism.

From the perspective of improving the quality of Chinese herbal medicines and standardizing the medical practices of TCM institutions, the implementation of a zero-markup policy for TCM herbal decoction pieces is increasingly proposed as a reform direction. Given the high dependence of TCM institutions on the markup policy for TCM herbal decoction pieces, directly implementing a zero-markup policy without corresponding fiscal subsidy policies and supporting medical insurance payment policies would subject these institutions to immense pressure on their survival and development, which could hinder the smooth progress of medical insurance payment method reforms.

Therefore, simply abolishing markups is not the end goal of the reform; the key lies in achieving a coordinated transition of supporting measures and medical insurance payment methods (18). To this end, relevant provincial-level departments should strengthen overall coordination, conduct scientific assessments, carefully study the issue, and introduce specific policies to create conditions for monitored implementation of reforms to abolish markups on TCM decoction pieces, while evaluating their effects on the sustainability of TCM medical institutions.

### 4.5 Shortcomings in quality assessment and the “higher quality, higher Price” mechanism amid centralized procurement of traditional Chinese medicine

In recent years, the proportion of traditional Chinese patent medicines in total drug expenditures has risen significantly. In response, the National Healthcare Security Administration has stated that centralized drug procurement is planned to be implemented across three sectors-chemical drugs, traditional Chinese patent medicines, and biologics-further expanding the scope of centralized procurement for traditional Chinese patent medicines. The full-scale advancement of centralized volume-based procurement of traditional Chinese medicines is intended not only as a cost-control measure but also as a preliminary condition for deepening the reform of healthcare payment methods for traditional Chinese medicine (19). In 2021, the General Office of the State Council issued the “Opinions on Promoting the Regularization and Institutionalization of Centralized Volume-Based Drug Procurement,” which proposed specific measures across five areas-scope, rules, safeguards, policies, and operational mechanisms-aimed at accelerating the formation of a unified and open national market for centralized drug procurement, reducing inflated drug prices, safeguarding the public’s access to essential medicines, and creating conditions for healthier development of the pharmaceutical industry. This initiative marked a policy step toward normalizing centralized volume-based drug procurement, subsequently bringing TCM patent medicines and TCM decoction pieces into the scope of the program. Under the national centralized procurement policy, the government directly engages in commercial negotiations with drug manufacturers with the stated aim of lowering prices while maintaining quality standards and enabling the public to access more affordable medicines. The core of payment method reform lies in establishing payment standards that align with actual costs. Prior to centralized procurement, prices for traditional Chinese medicines were inflated and volatile, leaving medical insurance programs without stable, reliable cost data when setting payment standards for specific diseases. The implementation of centralized procurement for proprietary Chinese medicines is intended to reduce price inflation and establish clearer, more unified drug cost benchmarks. This could help medical insurance authorities calculate and set reimbursement rates based on more realistic drug costs, but the rigor and sustainability of payment reform still depend on data quality, implementation capacity, and follow-up evaluation (20).

The main issues with centralized volume-based procurement of TCM products lie in the difficulty of standardizing quality standards for both TCM herbal materials and TCM products (21). Unlike chemical drugs, the quality of TCM products is influenced by multiple factors, including the quality of TCM decoction pieces, the formulations of proprietary Chinese medicines, and the manufacturing processes of TCM preparations. The quality of TCM decoction pieces is also affected by various factors such as the botanical origin of the herbal materials, the environmental conditions of their place of origin, the age of the plants, and processing and preparation methods, making it difficult to assess their quality based solely on a few simple indicators of ingredient content (22). Second, the costs of TCM products are unpredictable. Due to their unique properties, the prices of TCM herbal materials fluctuate frequently-particularly those of wild-harvested herbs. Force majeure factors such as climate and natural disasters can affect the supply and demand of TCM herbal materials, thereby impacting their quality, price, and cost. When medical insurance reimburses based on post-procurement costs, discrepancies in potential therapeutic value and expenses among enrolled cases due to variations in drug quality may lead to disputes regarding payment equity and healthcare quality. Therefore, promoting the establishment of a TCM quality evaluation system linked to clinical-value indicators is likely to be important for the coordinated advancement of centralized procurement and medical insurance payment reform.

**Figure 1:**
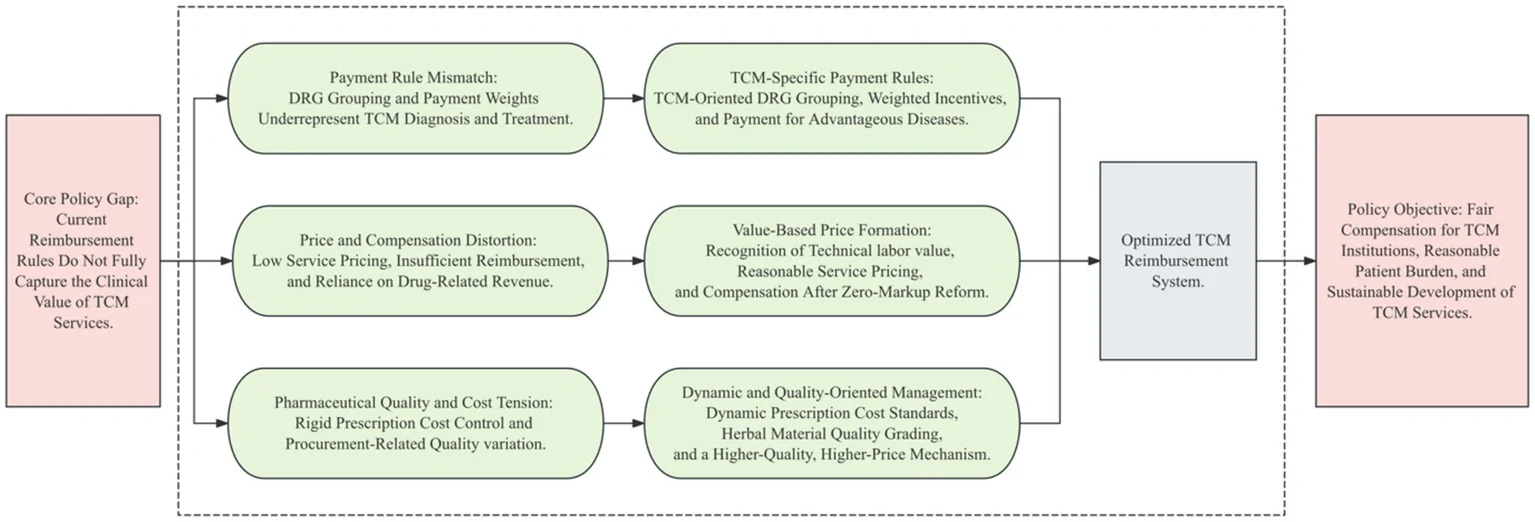
Core mechanism for aligning TCM reimbursement with clinical value.

To clearly illustrate the logical relationship between current limitations and policy optimization, Figure 1 presents a framework of the core mechanisms linking TCM medical insurance reimbursement policies to the clinical value of TCM. This framework suggests that current medical insurance reimbursement rules may not fully reflect the potential value of TCM services, indicating a need for coordinated optimization across three areas: reimbursement rules, price compensation, and quality management of Chinese herbal medicines.

## 5 Proposals for reforming health insurance payment methods for traditional Chinese medicine

As a medical science with a long history and a unique theoretical system, TCM urgently requires medical insurance reimbursement policies that align with its inherent principles to support its inheritance, innovation, and high-quality development. Currently, reforms to medical insurance payment methods-primarily centered on DRG-are advancing rapidly nationwide. However, because these reforms are designed primarily based on Western medical diagnostic and treatment models and coding rules, they do not yet fully align with the clinical characteristics and service models of TCM, which emphasize syndrome differentiation and treatment tailored to the individual, as well as holistic regulation. This may contribute to issues such as coding mismatches, imprecise grouping, and inadequate reimbursement for TCM medical institutions and specialized technical services, thereby constraining the conditions for an effective supply of TCM services and for realizing their distinctive features. To create conditions for the inheritance, innovation, and high-quality development of TCM within the context of medical insurance payment reform, there is an urgent need to establish a medical insurance payment and management mechanism that conforms to the principles of TCM and reflects its value.

### 5.1 Refine DRG grouping and payment rules tailored to traditional Chinese medicine

To address the current mismatch between the DRG payment system and the TCM diagnostic and treatment model-which makes it difficult to reflect the value of TCM treatment-efforts should be focused on developing disease grouping schemes tailored to the characteristics of TCM. The DRG medical insurance payment method originated abroad and was designed primarily based on Western medical diagnostic and treatment models and coding principles; it has not yet fully incorporated the distinctive features of TCM diagnosis and treatment or the corresponding coding rules. As a result, reimbursement standards for disease groups that align with TCM have not yet been fully refined. It is recommended that the Provincial Medical Insurance Bureau take the lead, in collaboration with the Provincial Health Commission, to jointly advance the reform of TCM medical insurance policies. At the same time, medical institutions should strengthen communication and collaboration with government regulatory authorities, proactively participate in research on clinical pathways for TCM-specific disease categories, and develop disease grouping schemes that are adapted to medical insurance management requirements while reflecting the distinctive features of TCM, so that they are both TCM-oriented and localized. Efforts should be accelerated to establish TCM diagnostic grouping rules and refine TCM diagnostic coding. With a focus on the inheritance and development of TCM, TCM conditions in which TCM is considered to have distinctive strengths and potential therapeutic value should be selected. In consultation with clinical experts and medical institutions, clear criteria for the cure of these conditions should be defined, TCM disease grouping should be refined and incorporated into medical insurance, and reasonable reimbursement policies should be formulated to create conditions for a medical insurance reimbursement system that aligns with the characteristics of TCM.

Assign point weights to secondary surgical procedures within the DRG system and refine the grouping criteria. To improve the alignment between medical surgical treatment services and the Western-origin DRG health insurance payment system, relevant government agencies need to reasonably assign point weights to secondary surgical procedures within the DRG system or introduce point bonuses. At the same time, it is crucial to further refine the grouping criteria to reduce improper practices by medical institutions-such as splitting hospital stays, fragmenting surgical procedures, and refusing to admit critically ill patients-which may be driven by operational pressures and could exacerbate tensions between doctors and patients. This measure aims to promote the rational provision of medical services, while effects on patient satisfaction and system development would require subsequent monitoring.

Strengthen the targeted nature of fiscal subsidies to promote the development of TCM hospitals and establish a parallel mechanism of “government subsidies and operational compensation.” The government should establish a dedicated fiscal fund and define clear compensation measures aimed at covering hospitals’ operating costs, with the aim of providing more stable support for public service areas such as infrastructure development, the cultivation of key disciplines, and the professional growth of TCM practitioners. Additionally, the preferential treatment for TCM services within the DRG medical insurance payment policy should be expanded. Based on the performance evaluation rankings of tertiary public TCM hospitals, greater support should be provided to TCM medical institutions within the region that meet transparent performance criteria, thereby incentivizing the provision of TCM services within these hospitals. When determining the extent of preferential treatment for TCM services, the TCM treatment rate can serve as a key basis for setting the preferential coefficient, and the TCM incentive coefficient within the DRG point-based payment system should be moderately increased. This approach is intended to help address the compensation challenges faced by TCM services under the DRG payment model, but its financial and service effects require monitoring.

### 5.2 Optimize the pricing mechanism tailored to the characteristics of traditional Chinese medicine and reform payment methods in the field of traditional Chinese medicine

To address the issues of unreasonable pricing for TCM services, the intensifying trend toward “Westernization” of TCM, and the fact that the current pricing mechanism lags behind actual demand, it is recommended that the Provincial Medical Insurance Bureau take the lead, in collaboration with the Provincial Health Commission, to reform medical insurance policies for TCM so that TCM services receive reasonable price compensation. A monitoring-oriented management mechanism for TCM service pricing items should be established; existing TCM pricing items should be optimized; and policies for managing the pricing of newly added TCM services should be improved. Localities should be encouraged to include appropriate TCM technologies-those with documented or clinically recognized suitability-within the scope of medical insurance coverage. For example, fees for the processing and dispensing of decocted Chinese herbal medicines should be added; new TCM medical service items should be encouraged; greater priority should be given to expanding medical insurance coverage; more Chinese herbal medicines should be included in the scope of reimbursement by the basic medical insurance fund; and reimbursement rates for TCM services could be adjusted where supported by cost and outcome evidence. For traditional TCM techniques derived from ancient classics that remain widely used today and have documented or clinically recognized suitability, as well as innovative new TCM technologies with potential economic advantages, the review procedures for new items should be streamlined, a fast-track process should be established, and such services should be included in medical insurance coverage.

At the same time, fully considering the distinct clinical characteristics of TCM hospitals compared to general hospitals, as well as their respective roles and positioning within the public health system, separate medical insurance fund pools should be established for each under the DRG medical insurance payment system. By reasonably allocating medical insurance funds and establishing separate medical insurance fund pools for TCM hospitals and general hospitals, a safety net and total budget management could be implemented for the share of medical insurance funds allocated to TCM hospitals. Payments to TCM hospitals and general hospitals could be made separately in accordance with their respective DRG medical insurance payment principles. At the same time, efforts could be advanced to explore and establish a set of detailed implementation rules for the DRG medical insurance payment system applicable to TCM hospitals. Health insurance authorities could promote a medical insurance payment model that combines a per-capita lump-sum allocation with DRG point-based payment, shifting incentives from maximizing points within a shared pooled budget to strengthening provider-level budget accountability, cost containment, prevention, and service optimization. They could also formulate policies based on patient health outcomes, shift the focus from service providers to patients, strengthen health insurance outcome management, establish population health evaluation indicators, and test whether the potential advantages of TCM preventive care and health management can be converted into incentives for retaining medical insurance fund surpluses. For TCM-advantaged conditions, policymakers could implement a therapeutic-value-based payment reform (23), enforce the principle of “same disease, same efficacy, same price” for both TCM and Western medicine, comprehensively evaluate TCM-advantaged conditions in both inpatient and outpatient settings, and explore a bundled payment model to unify revenue and payment mechanisms.

### 5.3 Dynamically adjusting the average cost control standards for traditional Chinese medicine prescriptions

To address the issues of the average cost standard for TCM prescriptions lagging behind rising prices and labor costs, failing to undergo dynamic adjustments, and becoming out of step with actual clinical needs, the management mechanism should be improved. Provincial health authorities should promptly publish the average cost standard for TCM prescriptions based on the annual price index and reduce direct restrictions on the number of herbal ingredients in TCM prescriptions. Given the changes in price indices and the rise in labor costs in recent years, raising the average cost control standard for TCM prescriptions could create conditions for more clinically appropriate prescription management and better alignment with public demand for TCM services, but its effects on clinical outcomes should be assessed through standardized monitoring indicators. In addition, moderately relaxing policy restrictions on TCM practitioners’ multi-site practice may support the mobility of TCM talent; however, effects on the quality of services provided by private medical institutions such as TCM clinics require further evaluation.

### 5.4 Prudently advance the reform to eliminate markups on TCM decoction pieces and implement supporting compensation measures

Given that the current markup policy for TCM decoction pieces may not have consistently achieved its intended compensatory function and may contribute to price increases, reforms to phase out this policy should be implemented in a prudent and measured manner. Drawing on the historical lessons from the markup policy for Western medicine, the elimination of the markup on TCM decoction pieces should be implemented gradually. Drawing on the experience of certain regions, the markup on TCM decoction pieces can be gradually eliminated or reduced based on scientific calculations of medical institutions’ revenue structures, material wastage, dispensing costs, and service costs. This transition should be supported through adjustments to medical service pricing, medical insurance reimbursement, and special fiscal subsidies.

The portion of the markup that has been eliminated could be used to compensate for the cost of technical services. A price supplementation mechanism could be established that is clinically value-oriented, focuses on TCM’s strengths and distinctive services, and is designed to better reflect the technical characteristics and potential value of TCM. This mechanism would aim to compensate for the revenue loss resulting from the elimination of the markup by raising the prices of TCM medical services. It is recommended that the Provincial Medical Security Bureau, in collaboration with the Provincial Department of Finance, reallocate the profits from the original markup through price adjustments to TCM service items or the pricing of priority outpatient conditions. Fees should be increased for TCM services such as “preventive medicine,” “TCM health literacy,” and constitution identification in health checkups, thereby better reflecting the technical value of TCM. Drawing on the approach taken after the elimination of markups on Western medicines, the prices of TCM medical service items could be appropriately raised or new items introduced. The relative pricing relationships among TCM service items should be optimized, establishing a price transfer mechanism where 80% is used to increase TCM medical service prices, 10% is covered by fiscal subsidies, and 10% is borne by the hospitals themselves.

### 5.5 Increase investment in the cultivation of traditional Chinese medicine and develop innovative quality grading standards for medicinal herbs

To address the limited observed effectiveness of centralized volume-based procurement of TCM, which may be affected by factors such as inconsistent quality standards and unpredictable costs, it is necessary to strengthen quality control at the source, increase investment in TCM cultivation, and develop innovative quality grading standards for TCM herbs. As a resource-intensive industry, both the “quality” and “quantity” of TCM herbs influence the development of the TCM industry and even impact the development of traditional Chinese medicine. Consequently, to better support the quality management of TCM herbs, policymakers can lead TCM enterprises in increasing investment in the cultivation sector, thereby aiming to support key agricultural sectors. By formulating development plans for the cultivation sector from the perspective of the downstream supply chain and rationally allocating cultivation resources based on the inherent growth characteristics of TCM herbs, policymakers can create conditions for more orderly development of the TCM herb cultivation industry and establish a supply system intended to improve both quality and quantity of TCM herbs.

Secondly, the TCM industry urgently needs to accelerate in-depth research and development to establish quality grading standards based on modern technologies and methods that can more systematically characterize the quality and potential clinical value of herbal medicines. Furthermore, “dao di” herbal materials are those that have been selected through long-term clinical application in traditional Chinese medicine. They are produced in specific regions, exhibit relatively strong quality attributes and clinically recognized value compared with the same species grown in other areas, and enjoy a high level of recognition. To promote a stable supply of high-quality herbal materials, it is also necessary to conduct in-depth research and formulate quality standards for these “dao di” herbal materials. This could help create conditions for a more stable supply system for herbal materials based on the principle of “higher quality, higher price,” while the effects on quality, prices, and industry development would still require evaluation.

## 6 Conclusion

Reforms to the medical insurance payment methods for TCM and the development of TCM services are potentially important components of policy efforts to develop TCM’s new-quality productive forces. They are intended to support the high-quality development of TCM and may provide policy guidance for its industrial upgrading and innovative development, while their actual effects require further evidence. The core of reforming TCM medical insurance payment methods lies in establishing a medical insurance payment and management mechanism that both respects the universality of Western medicine payment rules and reflects the unique characteristics of TCM diagnosis and treatment, thereby addressing key issues such as the localization of DRG, service pricing, markups on Chinese herbal medicines, and quality standardization. Through structured analysis of policy background and practical implementation, a systematic model and pathway can be proposed for further testing and refinement. This may serve as a reference for the Chinese government and other countries in further developing TCM services and advancing reforms in medical insurance payment methods; whether such reforms better meet the people’s health and preventive-care needs or contribute to broader health-system goals should be verified through implementation data and outcome evaluation.

## 7 Limitations

This study has several limitations. First, the analysis is based on publicly available policy documents and secondary literature rather than internal health insurance settlement records, hospital-level operational data, or patient-level outcome data. The findings therefore identify policy instruments and implementation pathways, but they should not be interpreted as estimates of causal effects on service utilization, patient access, medical expenditure, fund sustainability, or clinical outcomes. Second, Zhejiang is used as an illustrative focal case, and Shanghai, Guangdong, and Jiangsu are included as comparator regions through purposive policy sampling. The comparison captures variation in policy design but does not provide exhaustive national coverage. Third, because local implementation rules continue to evolve and publicly reported indicators are not standardized across jurisdictions, direct cross-regional quantitative comparison remains limited. Future research should combine policy analysis with claims data, provider surveys, patient-level utilization records, and longitudinal regional indicators to evaluate how TCM-specific payment reforms affect provider behavior, patient financial burden, quality of care, and health insurance fund performance.

These limitations also suggest caution in policy transfer. Measures such as TCM-specific DRG subgrouping, outpatient global-budget management, quality grading of herbal materials, and payment for TCM-advantaged diseases should be adapted to local service capacity, disease spectrum, fund affordability, and data governance conditions. Implementation should therefore combine TCM-oriented payment design with transparent monitoring indicators and periodic evaluation of access, quality, expenditure, and patient burden.

## Data Availability

The original contributions presented in the study are included in the article/supplementary material, further inquiries can be directed to the corresponding author.
